# Barriers and facilitators to the implementation of orthodontic mini-implants in clinical practice: a protocol for a systematic review and meta-analysis

**DOI:** 10.1186/s13643-016-0198-4

**Published:** 2016-02-05

**Authors:** Reint Meursinge Reynders, Laura Ronchi, Luisa Ladu, Nicola Di Girolamo, Jan de Lange, Nia Roberts, Sharon Mickan

**Affiliations:** Department of Oral and Maxillofacial Surgery, Academic Medical Center, University of Amsterdam, Meibergdreef 9, 1105 AZ Amsterdam, The Netherlands; Private practice of orthodontics, Via Matteo Bandello 15, 20123 Milan, Italy; Department of Veterinary Sciences, University of Bologna, Via Tolara di Sopra 50, 40064 Ozzano dell’Emilia (BO), Italy; Department of Oral and Maxillofacial Surgery, Academic Medical Center and Academisch Centrum Tandheelkunde Amsterdam (ACTA), University of Amsterdam, Meibergdreef 9, 1105 AZ Amsterdam, The Netherlands; Bodleian Health Care libraries, John Radcliffe Hospital, University of Oxford, Cairns Library Level 3, Oxford, OX3 9DU UK; Department of Allied Health, Clinical Governance, Education and Research, Gold Coast Health Griffith University, Executive Offices A Block Level 4. 1 Hospital Blvd, Southport, QLD 4215 Australia

**Keywords:** Mini-implant, Screw, Orthodontics, Implementation, Knowledge translation, Barriers, Facilitators, Contacting authors, Systematic review

## Abstract

**Background:**

Most orthodontic treatment plans need some form of anchorage to control the reciprocal forces of tooth movement. Orthodontic mini implants (OMIs) have been hailed for having revolutionized orthodontics, because they provide anchorage without depending on the collaboration of patients, they have a favorable effectiveness compared with conventional anchorage devices, and they can be used for a wide scale of treatment objectives. However, surveys have shown that many orthodontists never or rarely use them. To understand the rationale behind this knowledge-to-action gap, we will conduct a systematic review that will identify and quantify potential barriers and facilitators to the implementation of OMIs in clinical practice for all potential stakeholders, i.e., patients, family members, clinicians, office staff, clinic owners, policy makers, etc. The prevalence of clinicians that do not use OMIs will be our secondary outcome.

**Methods:**

The Preferred Reporting Items for Systematic review and Meta-Analysis Protocols (PRISMA-P) 2015 Statement was adopted as the framework for reporting this manuscript. We will apply broad-spectrum search strategies and will search MEDLINE and more than 40 other databases. We will conduct searches in the gray literature, screen reference lists, and hand-search 12 journals. All study designs, stakeholders, interventions, settings, and languages will be eligible. We will search studies that report on barriers or facilitators to the implementation of orthodontic mini implants (OMIs) in clinical practice. Implementation constructs and their prevalence among pertinent stakeholders will be our primary outcomes. All searching and data extraction procedures will be conducted by three experienced reviewers. We will also contact authors and investigators to obtain additional information on data items and unidentified studies. Risk of bias will be scored with tools designed for the specific study designs. We will assess heterogeneity, meta-biases, and the robustness of the overall evidence of outcomes. We will present findings in a systematic narrative synthesis and plan meta-analyses when pertinent criteria are met.

**Discussion:**

Knowledge creation on this research topic could identify and quantify both expected and unexpected implementation constructs and their stakeholders. Such knowledge can help develop strategies to address implementation issues and redirect future studies on OMIs towards knowledge translation. This could lead to improved patient-health experiences and a reduction in research waste.

**Electronic supplementary material:**

The online version of this article (doi:10.1186/s13643-016-0198-4) contains supplementary material, which is available to authorized users.

## Background

The introduction of a new technique into a health-care system is a complex process, depends on the successful interaction between a variety of stakeholders, but often fails [1–4]. This failure is a global problem and has created a knowledge-to-action (KTA) gap, which is the gap between evidence-based knowledge and the use of this information in practice [5]. This gap also applies to orthodontic mini-implants (OMIs), because the implementation of this health technology into the clinical practice is often suboptimal [6–9]. To address this issue, it is important to identify and quantify barriers and facilitators to the implementation of OMIs. This systematic review will focus on these objectives.

**Fig. 1:**
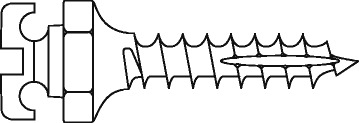
Schematic of an orthodontic mini-implant

Orthodontists use various types of anchorage to control the reciprocal forces of tooth movement. Anchorage is necessary in most orthodontic treatment plans and is usually obtained by applying forces to groups of teeth or through extra-oral sources, for example, the neck or cranium [10]. However, these techniques pose serious limitations such as (1) their restricted area of application, (2) they may still cause loss of anchorage, and (3) they depend on the collaboration of the patient [10]. OMIs are not conditioned by most of these shortcomings, are indicated for a wide variety of treatment mechanics, and can be used in both jaws over long time periods [11] (Fig. 1). These advantages should be considered in the context of some of limitations of these devices such as (1) the need to conduct surgery for their placement; (2) risk factors associated with surgery; (3) implant failure and its implications; (4) costs; and (5) numerous implementation issues. Conducting surgical interventions in orthodontic offices is still very uncommon. Implementation can be conditioned by variables such as (1) the lack of knowledge and skills of clinicians to conduct such interventional procedures; (2) the lack of knowledge-management skills of pertinent stakeholders; (3) lack of an adequate organization; (4) lack of time and resources; (5) attitudes towards new knowledge; (6) perceptions of various stakeholders regarding the quality and validity of the evidence on OMIs; (7) resistance within the organization; and (8) resistance from the patient [5].

**Fig. 2:**
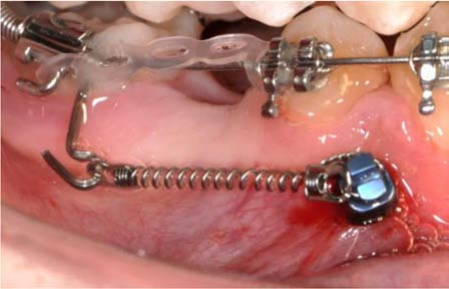
An orthodontic mini-implant* immediately after insertion and orthodontic loading. *Quattro implants PSM Medical Solutions; Tuttlingen, Germany

The interventional procedure can be divided in four phases: (1) implant insertion; (2) orthodontic loading; (3) implant maintenance; and (4) implant removal. In the first phase, specific OMIs are selected according to the indicated anchorage objectives. Machine surfaced OMIs with a diameter of 1.3–2 mm and a length of 6–10 mm are currently the most frequently used (Fig. 1) [12]. After the administration of a local anesthetic, OMIs are inserted into either the maxillary or mandibular bone. Immediately after insertion, they are generally loaded with light orthodontic forces of 100 g (Fig. 2). The first appointment of the maintenance phase is programed 1 week after implant insertion. Patients are subsequently checked every 4 weeks. During these maintenance visits, appliances and orthodontic forces are controlled, implant stability is assessed with an explorer, and implant maintenance-related factors are reinforced. At the completion of all anchorage objectives, OMIs are removed, usually without the need of anesthesia. Insertion sites generally heal without complications.

**Fig. 3:**
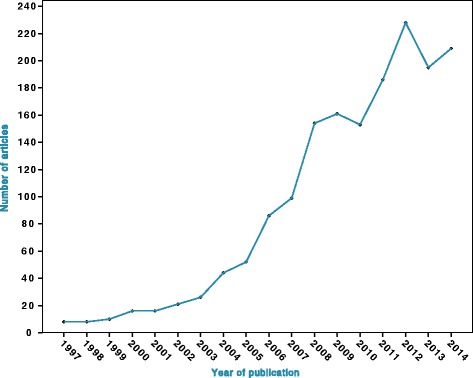
Scatter plot of the number of articles on OMIs identified per year since 1997 in Pubmed (MEDLINE) $. $ Search strategy in PubMed: ((orthodont* AND (mini implant* OR micro implant* OR microimplant* OR screw* OR mini screw* OR miniscrew* OR micro screw* OR microscrew* OR temporary anchorage device*))

OMIs have been hailed for having revolutionized clinical orthodontics, and systematic reviews have reported low implant failure rates and have recorded favorable effectiveness of OMI reinforced anchorage compared with anchorage obtained with conventional treatment mechanics [11–14]. OMIs are among the most frequently presented topics at orthodontic meetings, and a 4-day world implant orthodontic conference is organized annually with OMIs as its exclusive topic [15]. In addition, the number of publications on OMIs has increased exponentially since the introduction of OMIs by Kanomi in November 1997 (Fig. 3) and numerous orthodontic implant companies have been founded [16, 17].

However, surveys in a variety of countries have shown that many orthodontists never or rarely use these devices [6–9, 18]. This KTA gap is a surprise for a technique that was introduced almost 20 years ago and that has the advantageous characteristics described previously [11–13, 16]. This is further surprising, because anchorage systems are indicated in most orthodontic treatment plans [10]. A recent survey showed that more than 75 % of the surveyed doctors would include OMIs in a treatment plan for a common orthodontic patient [19].

To understand the rationale behind the KTA gap, it is necessary to identify and quantify barriers and facilitators to the implementation of OMIs (the implementation constructs) for both demand-side stakeholders, i.e., orthodontic patients and their family members and potential supply-side stakeholders, e.g., clinicians, office staff, clinic owners, researchers, guideline developers, policy makers, implant companies, etc. Barriers to implementation could include the invasiveness of the procedure, learning a new technique, fear of complications, financial obstacles, the large volume of research evidence, the lack of trust in research evidence, the applicability of the new health technology to a local context, etc. [6, 8, 20–22]. Facilitators to implementation could include shortened treatment time, better outcomes, improved esthetics during orthodontic treatment, recommendations by patients who had undergone treatment with OMIs, etc. [6, 23, 24]. Identification and quantification of these barriers and facilitators are important, because knowledge gaps can be subsequently assessed and strategies can be developed to deal with them. For our secondary objectives, we will record the prevalence of clinicians that do not use OMIs in the eligible studies of our primary question. This statistic quantifies the knowledge-to-action gap for these stakeholders. Our scoping searches showed that no earlier systematic review or protocol has addressed the objectives of this systematic review protocol.

## Objectives

The objectives of this systematic review are as follows:

### Primary objectives

- To identify and quantify barriers and facilitators to the implementation of OMIs for all potential stakeholders such as patients and their family members, clinicians, office staff, clinic owners, researchers, guideline developers, policy makers, implant companies, etc.

### Secondary objectives

- To record the prevalence of clinicians that does not use OMIs in the studies that will be selected for the primary objectives.

## Methods

To develop our methods section, we consulted (1) conceptual models for assessing barriers and facilitators to knowledge use [20, 25–28]; (2) guidelines and handbooks for conducting quantitative and qualitative systematic reviews [29–32]; (3) checklists for reporting research studies [33]; (4) systematic reviews that were developed to identify barriers and facilitators on a variety of issues and asked similar research questions as our research study [2, 21, 22, 34–37]; and (5) our previous systematic reviews and a protocol of a systematic review on OMIs [38–41].

We adopted the Preferred Reporting Items for Systematic review and Meta-Analysis Protocols (PRISMA-P) 2015 statement as the guideline for reporting this protocol [42, 43]. This manuscript is not registered in PROSPERO, because our research questions are not covered by the inclusion criteria of this register [44].

### Eligibility criteria

#### Study designs

For the primary objectives, we defined the following eligibility criteria:

- To maximize the breadth of data collection, studies will not be excluded based on their research design. Studies that present an original collection of data on identified barriers and facilitators to the use of OMIs will be eligible. Such studies generally refer to interviews, focus groups, surveys, and questionnaires with any of the pertinent stakeholders [22].
- Studies that identify or quantify barriers and facilitators to knowledge use as a primary or as an additional objective of a larger study will be both eligible. For example, a study that addresses our research questions nested in a larger research model such as mixed methods will also be eligible.

For the secondary objectives, we defined the following eligibility criteria:

- Only quantitative studies, for example, surveys, that addressed the primary objectives of this systematic review will be eligible.

### Stakeholders (participants)

Barriers to implementation can vary for different stakeholders. To avoid inappropriate exclusion of pertinent barriers, we will apply broad-spectrum eligibility criteria that include any possible demand and supply side stakeholders. The former group refers to orthodontic patients and their family members. The latter stakeholders refer to clinicians, office staff, clinic owners, researchers, guideline developers, policy makers, implant companies, etc.

### Interventions

Interventions that use one or more implants with diameters smaller than 2.5 mm for orthodontic anchorage purposes will be eligible. No restrictions will be applied to the implant length or design, the connection to plates, the location of insertion, the type of insertion procedure, the type of orthodontic loading, and the type of implant maintenance. Interventions with OMIs on patients of either sex, and in any age or demographic group will be eligible.

### Outcomes

Any type of barrier or facilitator to the use of OMIs in clinical practice will be our primary outcome. A barrier is defined as any variable that impedes or obstructs their use. A facilitator is defined as any variable that eases and promotes the use of OMIs. Examples of barriers and facilitators were presented in the introduction. Barriers and facilitators are eligible as outcomes when they are described as implementation constructs by the eligible stakeholders [37]. For example, patient’s perceptions of the interventional procedure or assessments of health experiences such as pain and discomfort during implant insertion will not be considered as eligible outcomes when they are not specifically defined as barriers to the use of OMIs by these patients. These eligibility criteria avoid mislabeling of implementation constructs during qualitative analyses as a result of bias or misinterpretation of outcomes by systematic reviewers.

### Setting and language

No setting and language restrictions will be applied.

### Information sources

Information sources will be searched from January 1 1997, the year of the first publication on OMIs, onwards [16]. We adopted a variety of information sources from previous systematic reviews on OMIs [38–41]. TRIP and SUMSearch databases were also consulted to identify pertinent search engines [45, 46].

### Electronic searches

- The following general and subject-specific electronic databases will be searched: Google Scholar Beta, PubMed (MEDLINE), EMBASE (Ovid), Cochrane Central Register of Controlled Trials (CENTRAL), CINAHL, PsycINFO, Sociological Abstracts, and PROSPERO [47–52].
- The ‘Related Articles’ feature in PubMed will be consulted.
- The following Web of Science Core collection citation indexes will be searched: Science Citation Index Expanded (SCI-EXPANDED); ARTS & Humanities Citation Index (A&HCI); and Social Sciences Citation Index (SSCI) [49, 53, 54].
- A series of national and regional databases will also be searched: African Index Medicus, African Journals online (AJOL), Informit Health Collection, Index Medicus for the Eastern Mediterranean Region, IndMED, KoreaMed, LILACS, Index Medicus for the South-East Asia Region (IMSEAR), and Western Pacific Region Index Medicus (WPRIM) [49, 53].

### Searching other resources

#### Gray literature

We will consult the following gray databases [49, 50, 53, 55]:

- General databases: Open Grey, Google Scholar Beta, The National Technical Information Service (NTIS), and The Health Management Information Consortium (HMIC).
- Dissertations and databases of theses: ProQuest and Dissertations and Theses (Global full text plus UK and Ireland abstracts).
- Conference proceedings and abstracts: Meeting Abstracts, ISI Conference Proceedings, IEEE Conference Proceedings, and Google Scholar.
- Review databases: Database of Abstracts of Reviews of Effects (DARE), Health Technology Assessment database (HTA), NHS EED all via the Center for Reviews and Dissemination (CRD), and Turning Research into Practice (TRIP).
- Guidelines will be searched in MEDLINE, EMBASE, and TRIP. In addition, evidence-based guidelines of the following organizations will be consulted: Australian National Health and Medical Research Council, Canadian Medical Association, National Guideline Clearinghouse, National Library of Medicine Guidelines, New Zealand Guidelines Group, and NICE Clinical Guidelines.
- We will search Pubmed and Ovid for Citation alerts.

#### Handsearching

- *American Journal of Orthodontics & Dentofacial Orthopedics, Angle Orthodontist, Australian Journal of Orthodontics, European Journal of Orthodontics, International Journal of Adult Orthodontics and Orthognathic surgery, Journal of Clinical Orthodontics, Journal of Orthodontics, Journal of the World Federation of Orthodontics, Orthodontics & Craniofacial Research, and Seminars in Orthodontics.*

#### Reference lists

- We will also manually screen the reference lists of all selected articles, reviews, and guidelines for eligible papers that are not identified during the other searching procedures.

#### Correspondence

- A variety of stakeholders, e.g., subject specialists, authors of selected papers and pertinent systematic reviews, researchers on OMIs and manufacturers of implants, will be contacted to identify ongoing or unpublished research studies [53, 56].

### Search strategy

- Methods to find pertinent subject headings and keywords are adopted from our previous systematic reviews on OMIs [38–41]. For the electronic database searches, we will use variations of search terms for the field of interest (orthodontics) and the intervention of interest (mini implants). To avoid the inappropriate exclusion of pertinent studies, we will not include ‘the outcomes of interest’ as selection criteria and will aim at a broad-spectrum search strategy. Search terms are identified in collaboration with an information specialist (NR) and include orthodontic(s), orthodontist(s), implant(s), mini implant(s), micro implant(s), microimplant(s), screw(s), mini screw(s), miniscrew(s), micro screw(s), microscrew(s), and temporary anchorage device(s).
- Search strategies will be developed specifically for each database and will be subsequently pilot tested and fine-tuned [53].
- An information specialist (NR) will assist with the development of these search strategies.
- To avoid the incorrect exclusion of eligible studies, the Boolean ‘NOT’ operator will not be used.
- The search strategies of all general and subject-specific electronic databases will be listed in a table together with the search dates and the number of identified items. Examples of the search strategy of MEDLINE and Google Scholar Beta are presented in Table 1 [49, 57].

**Table 1:** Search strategy for the MEDLINE and Google Scholar Beta databases

PubMed (Medline)	orthodont* AND (implant* OR mini implant* OR micro implant* OR microimplant* OR screw* OR mini screw* OR miniscrew* OR micro screw* OR microscrew* OR temporary anchorage device*)
Google scholar beta^a^	(orthodontics OR orthodontic OR orthodontist OR orthodontists) (implant OR implants OR “mini implant” OR “mini implants” OR screw OR screws OR “mini screw” OR “mini screws” OR “miniscrew” OR “miniscrews” OR “microscrew” OR “temporary anchorage device”)

For each database, we will adapt the pertinent characters for the exploration and truncation of the search terms. All search strategies will be copied and pasted directly into the search box of the search engines. This procedure will be applied, because re-typing of search terms could introduce errors in the search strategy [49]

^a^We will use this shortened search strategy because the search string of Google Scholar is limited to under 256 characters [57]

## Study records

### Data management

To reduce inter-examiner disagreements on study eligibility, we will adopt the procedures described in the Cochrane Handbook for Systematic Reviews of Interventions and in the PRISMA-P 2015 statement [42, 43, 58]. Prior to starting the formal study selection process, we will pilot test our selection procedures on a sample of abstracts. These calibration procedures are conducted to clarify and potentially fine-tune our selection criteria and to apply them consistently. All three review authors (RMR, LR, and LL) will participate in these calibration exercises.

### Selection process

- We consulted the study selection procedures of our previous systematic reviews on OMIs to develop this section [38–41]. We will select studies that fulfil our eligibility criteria.
- Studies will be selected independently by three experienced systematic reviewers (RMR, LR, and LL), who are also topic experts.
- We will screen titles and abstracts for eligible studies. Each selected abstract will be linked to the data source of origin. Full texts of potentially relevant articles will be subsequently reviewed. To reduce the risk of inappropriate exclusion, ambiguous articles will also be assessed for eligibility.
- Unpublished research studies, e.g., those found in gray literature databases, will also be reviewed for eligibility by the three reviewers. Such studies are only considered when sufficient data are reported to permit peer-reviewing.
- Authors will be contacted when potential multiple publications of their research data are identified. We will apply our protocol for contacting authors that we described in a recent protocol for a systematic review [40]. Characteristics of studies suspect of multiple publications include the following: (1) studies with a retrospective research design and that use similar methodology; (2) overlapping authors in similar research studies and placed in a different order; (3) the publication of similar research findings within a short time span in different journals; and (4) reference lists of the multiple publication articles tend to exclude the references of the other similar publications [40].
- Disagreements between authors on eligibility will be resolved through discussions. Persisting disagreements will be addressed through consultations with a fourth author (NDG) or through our protocol for contacting authors [56, 59]. A detailed description of our protocol for contacting authors is described in Additional file 1 and is based on one of our recent systematic review protocols [40]. The Cochrane Glossary will be consulted to avoid misinterpretation of terminology used in the email correspondence with authors [60].
- Study selection procedures will be presented in a PRISMA flow diagram [56, 61]. Excluded studies together with the rationale for their exclusion will be listed in a table.

### Data collection process

- Prior to the formal study selection and data extraction process, a list of ‘potential’ barriers and facilitators to the implementation of OMIs will be developed. Items on this list will be extracted from three groups of publications: (1) systematic reviews that focussed on the identification of barriers and facilitators to the implementation of health-related issues and technologies [2, 21, 22, 34–37]; (2) conceptual models for assessing barriers and facilitators to knowledge use [20, 25–28]; and (3) our previous systematic reviews on OMIs [38–41].
- These articles will be examined by all three reviewers (RMR, LR, and LL), which are all topic experts. Each of these operators will develop a list of barriers and facilitators based on the findings in these publications that could be pertinent to the implementation of OMIs. These lists will be subsequently discussed between these three reviewers and a final summary list of ‘potential’ barriers and facilitators will be created. We will also link specific stakeholders, e.g., patients, clinicians, office staff, etc., to each of these variables. In this list, ‘potential’ barriers and facilitators are presented as a series of constructs and are classified according to five domains: intervention characteristics, outer setting, inner setting, characteristics of the individuals involved, and the process of implementation [25]. We adopted this classification from the Consolidated Framework For Implementation Research (CFIR) [25].
- Our procedures for the identification of ‘potential’ barriers and facilitators will be conducted prior to the study selection and data extraction process and are only used as a calibration exercise for the three reviewers and to increase their background knowledge. Our list of ‘potential’ barriers and facilitators will not be used as a reference checklist during the study selection and data collection process, because this could result in the inappropriate exclusion of ‘unexpected’ barriers and facilitators to the implementation of OMIs in clinical practice.
- For the development of our data extraction forms, we explored the reporting checklists of pertinent research designs of the equator network [33]. We also consulted data collection forms in previous systematic reviews on OMIs [38–41] and the three groups of publications that were used to develop the list of potential implementation constructs. Pertinent items for the extraction of data for the secondary research question were also explored during this research phase.
- Data extraction forms were first pilot tested on a series of articles by three reviewers (RMR, LR, and LL) and subsequently fine-tuned. These procedures were also used to calibrate reviewers. The pilot-tested data extraction forms are presented in Additional file 2 [43].
- Data extraction procedures will be conducted independently by the three aforementioned operators, who are experienced systematic reviewers and topic experts. Disagreements on extracted items will be resolved through rereading and discussions and if necessary an arbitrator (NDG) will be consulted to adjudicate these disagreements. All data extraction procedures will be similar for our primary and secondary research questions.

### Data items

- We decided to extract a broad spectrum of data items and define them in great detail, because revisiting all selected papers as a result of inaccurate or deficient listings of data items is a waste of ‘reviewers’ time and could introduce mistakes [43].
- Extracted data items for our primary and secondary objectives include the following: the source, eligibility, duplicate publication, the study design, selection procedures, stakeholders, the setting, interventions, outcomes, flow and timing, adverse effects, withdrawals and missing outcomes, the funding, and miscellaneous data of the selected studies [42, 43]. Many of these items are further subdivided and all extracted entries are listed in tables in Additional file 2. Descriptions of each item are presented in these tables and entries that could bias the outcomes are also recorded.
- Diagrams will be created to depict the flow of the stakeholders and the timing of the various research phases from the start of the selection procedures to the completion of the recording of outcomes (Additional file 2).
- Data from articles that appear ‘suspect’ of multiple publications of the same research studies will be extracted identically as those from articles that are not suspect. Potential overlap of research data will be subsequently assessed.
- When during the review process new relevant data items will be identified, we will collect them and will report these changes of the protocol and their rationales.
- Authors of selected studies will be contacted to find information on unclear or missing items and to resolve remaining disputes between reviewers (Additional file 1). Persistent disagreements will be reported.

## Outcomes and prioritization

### Primary outcomes

- The primary outcomes will be all barriers and facilitators to the implementation of OMIs in clinical practice identified by all demand- and supply-side stakeholders. We will record the prevalence of these implementation constructs among pertinent stakeholders.
- Pertinent stakeholders are defined in Table 2 and are further subdivided in ‘users’ and ‘non users’. A barrier is defined as any variable that impedes or obstructs the use of OMIs. A facilitator is defined as any variable that eases and promotes their use.

**Table 2:** Potential subgroups for which outcomes are recorded

General subgroups	Specific subgroups
Stakeholders	*Demand-side stakeholders:* orthodontic patients and their family members
	*Supply-side stakeholders:* clinicians, office staff, clinic owners, researchers, guideline developers, policy makers, implant companies, etc.
	*Users/non users*: both demand- and supply-side stakeholders can be further subdivided in those that have used OMIs previously (users) and those that do not use these devices (non-users)
Interventions	*Specified interventions:* these interventions refer to a specific phase or type of the interventional procedure. Phases of the intervention refer to the: anesthetics, implant insertion, orthodontic treatment with OMIs, implant removal, or the healing phase. Types of interventions refer to the implant type and dimensions, number of implants, use of plates, the surgical procedure, implant location, timing and forces of orthodontic loading, etc. [41].
	*‘Non specified’ interventions:* these interventions refer to ‘any orthodontic treatment with OMIs’. Additional information on the specific phase or type of the interventional procedure is not provided
Time points	*Pre-intervention recordings*, i.e., recordings of outcomes prior to the interventional procedure
	*Immediate post-intervention recordings*, i.e., recordings of outcomes within 2 weeks after the completion of the interventional procedure
	*Long-term post-intervention recordings*, i.e., recordings of outcomes more than 2 weeks after the completion of the interventional procedure.
Setting/Country	*Private practice:* stakeholders treated or working in a private practice
	*University setting:* stakeholders treated or working in a university clinic Country: stakeholders treated or working in a specific country
Research design	Surveys or questionnaires: outcomes obtained through either surveys or questionnaires
	Interviews: outcomes obtained from interviews with stakeholders
	Focus groups: outcomes obtained from focus groups with stakeholders
- All primary outcomes will be presented with an explicit description according to the author(s) of the pertinent eligible study. We will report whether these outcomes refer to ‘specified’ or ‘non-specified’ interventions (Table 2).
- When studies record our primary outcomes at different time points, we have decided a priori to subdivide them as pre-, immediate post-, and long-term post-intervention recordings (Table 2). We will also present the setting, country, and design of the research study.
- The pre-intervention recording of barriers or facilitators to a ‘specified’ intervention with OMIs for ‘non user’ clinicians or for patients that have not undergone this intervention previously in any type of setting or research design will be our ‘preferred’ primary outcome.
- The prevalence of identified barriers and facilitators among the surveyed or interviewed pertinent stakeholders will be calculated as follows:

Prevalence of an identified barrier or facilitator =

The number of stakeholders that scored a particular construct as a barrier or facilitator to the implementation of OMIs in clinical practice/The total number of stakeholders that scored on the role of this particular construct as a barrier or facilitator to the implementation of OMIs in clinical practice

This prevalence will be presented for example as: 30/50

- ‘Characteristics and findings of included studies’ tables are presented in Additional file 3. These tables list barriers and facilitators to the implementation of OMIs (implementation constructs), prevalence statistics, and other pertinent items such as the type of research design, stakeholders, settings, interventions, and outcomes, and time points for recording them. The procedures to extract and categorize primary outcomes and anticipated exemplary tables of categorized implementation constructs are also presented (Additional file 3) [43].

### Secondary outcomes

- The secondary outcomes will be the prevalence of clinicians that do not use OMIs and represent the knowledge-to-action gap. This statistic will be calculated as follows:

The prevalence of clinicians that do not use OMIs =

The number of clinicians that do not use OMIs/ The total number of surveyed clinicians that reported on the use of OMIs in clinical practice

Information that could give further insights in the understanding of the knowledge-to-action gap, e.g., the number of implants placed per clinician per year will also be recorded.

### Risk of bias in individual studies

The methodological quality of each eligible study will be assessed with critical appraisal tools that are specific for the type of research design used in that study. For qualitative studies such as focus groups and interviews, we will use the Joanna Briggs Institute Qualitative Assessment and Review Instrument (JBI QARI) [30]. For quantitative studies such as surveys and questionnaires, we will use the Joanna Briggs Institute critical appraisal tool for studies reporting prevalence and incidence data [31, 35, 62]. These instruments are listed in ‘Additional file 4’. The guidelines for scoring these tools will be first examined by the three reviewers (RMR, LR, and LL) [30, 31, 62, 63]. A series of studies will be then used to calibrate these reviewers for each appraisal tool. Eligible studies will be subsequently scored independently by these authors. To facilitate the comparisons of appraisal scores, all three authors will record the rationale for each of these scores and the location in the article. Initial critical appraisals are conducted during the data extraction phase of each eligible study and are revisited and fine-tuned after the completion of these procedures for all selected studies. Scoring differences between reviewers are resolved through discussions. A fourth reviewer (NDG) is called upon in the case of disagreement between reviewers. Authors of eligible studies are contacted in the case of persistent disagreements on appraisal scores (Additional file 1).

The critical appraisal scores for each selected study will be listed in tables and for each appraisal tool separately (Additional file 4) [30, 31]. We will calculate the prevalence of ‘Yes’ scores (number of ‘Yes’/number of articles) for each individual appraisal question [35]. No attempts will be made to calculate the overall appraisal scores. The potential influence of each of the scored answers on the outcomes of each selected study will be weighted during the data synthesis and will be used to assess the overall strength of evidence of the review (See ‘Confidence in cumulative evidence’) [43].

## Data synthesis

### Criteria for a quantitative synthesis

The prevalence statistics of our primary and secondary outcomes can both be synthesized quantitatively. A random-effects model meta-analysis will be indicated, because between study variance is expected for both outcomes. However, we will only conduct a meta-analysis when (1) the risk of bias in the eligible studies is low; (2) outcomes are consistent between studies; (3) publication bias is low; (4) a high number of studies is included; and (5) heterogeneity is low [64–66].

### Summary measures for a quantitative synthesis

The prevalence data for our primary outcomes will be presented as event rates, e.g., 0.70, which indicate that 70 stakeholders scored a particular construct as a barrier to the implementation of OMIs out of a total sample of 100 stakeholders that scored on the role of this particular construct as a barrier to the implementation of OMIs in clinical practice. Event rates will also be recorded for our secondary outcomes, which represent the number of clinicians that do not use OMIs/the total number of surveyed clinicians that reported on the use of OMIs in clinical practice. These statistics are quantitatively synthesized and the summary event rate will be calculated and presented with the *p* value and the 95 % confidence interval (CI) for both primary and secondary outcomes. Comprehensive Meta-analysis (CMA) software will be used to conduct all statistical analyses in this systematic review [67, 68]. Forest plots will be used to display these calculations and their dispersion.

### Unit-of-analysis issues for a quantitative synthesis

To deal with unit-of-analysis issues, we will assess at which level randomization was conducted [65]. We will assess whether all participants underwent the same intervention, multiple interventions, and whether outcomes were recorded at different or multiple time points [65]. Subgroups will be created to deal with these issues (see ‘Subgroup analyses and meta-regression’ section).

### Dealing with missing data for a quantitative synthesis

We will apply the protocol of the Cochrane Handbook for Systematic Reviews of Interventions for dealing with missing data [69]. We will first contact the pertinent authors to obtain such data (Additional file 1). We will then evaluate why data are missing and will assess whether they are missing at random or not [69]. Our key policy will be to include studies with missing data and assess the consequences of this inclusion in the qualitative synthesis [69]. For the quantitative synthesis, we will weigh the following strategies for dealing with missing data, i.e., (1) analysing available data only; (2) imputing the missing data; and (3) perform sensitivity analyses [69]. We will assess these strategies in the results and discussion section of our systematic review and will discuss the potential implications for excluding missing data from the meta-analysis [69].

### Investigation of heterogeneity

We will consider three sources of heterogeneity: methodological, clinical, and other sources of heterogeneity [65, 70]. These sources are selected a priori based on information from previous systematic reviews on this research topic and through discussions between the reviewers, who include both methodologists and topic experts [38–40, 71]. These potential sources of heterogeneity are listed in Additional file 5 [41, 65, 66, 70, 72–74]. The type of stakeholders, i.e., patients, clinicians, office staff, etc., is excluded as a source of diversity, because outcomes are analyzed separately for each type of stakeholder. We will report when ‘post hoc’ defined sources of heterogeneity will be investigated.

Statistical heterogeneity is the consequence of one or more of the sources of diversity. The presence of statistical heterogeneity is investigated by calculating the Cochran’s Q, the degrees of freedom based on the number of eligible studies, and the pertinent *p* value [75–77]. We will also calculate the following statistics: Kendall’s Tau^2^, Tau, and *I*^2^ [68, 75, 77–80]. These calculations, their use, and strategies for dealing with heterogeneity are explained in Additional file 5 [65].

### Subgroup analyses and meta-regression

Subgroup analyses are either used to explore heterogeneity or to address questions about specific stakeholders, interventions, or study designs [65]. For the former objective, we will use the subgroups defined under ‘methodological, clinical, and ‘other’ sources of heterogeneity in Additional file 5. For the latter objective, we will assess the following ‘a priori’ subgroups, which could be used for either subsets of participants or studies [65]:

- *Research design*, i.e., surveys or questionnaires, interviews, or focus groups
- *Conduct and analysis of the study*, i.e., high versus low risk of selection, performance, detection, attrition, or reporting bias [66].
- *Stakeholders*, i.e., ethnicity, sex, age, and previous experience with OMIs.
- *Type of interventions*, i.e., ‘specified’ or ‘non specified’ interventions (Additional file 5).
- *Outcomes*, i.e., pre-, immediate post-, or long-term post-intervention recordings of outcomes (Additional file 5).
- *Setting*, i.e., private practice or university setting.

Rationales to conduct analyses with additional subgroups will be reported in the systematic review and these subgroups will be defined as ‘post hoc’. Random effects meta-regression will be used to assess differences in subgroups, but will be only conducted when there are more than ten eligible studies in the meta-analysis [65]. We will interpret subgroup analyses and meta-regressions with caution, because these analyses are not based on randomized comparisons and are therefore strictly observational [65, 81]. Additional caveats of these analyses as defined by Oxman and Guyatt will also be considered [81].

### Sensitivity analysis

We will conduct sensitivity analyses to assess the impact on outcomes of certain decisions that were made during the systematic review process. We will plan such analyses for the decisions to include the following: (1) small studies (compared to the other eligible studies); (2) surveys and questionnaires; (3) gray literature; (4) studies at high risk of bias; and (5) studies with follow-ups beyond 6 months [65]. We will also conduct sensitivity analyses to assess the effects of imputing missing data [65, 69].

### Qualitative synthesis

We will provide a systematic narrative synthesis even when quantitative analyses are possible [43]. Our narrative synthesis will be conducted systematically and transparently to reduce the potential for bias [82]. We will refrain from vote counting, i.e., counting those studies that yielded a significant result and those that did not [83, 84]. As suggested by the PRISMA-P 2015 statement [43], we will adopt the ‘established methods’ for conducting systematic narrative syntheses according to the guidance of the CRD [82]. The CRD framework for conducting such a synthesis consists of four phases: (1) developing a theory why and how each barrier or facilitator could affect the implementation of OMIs for each linked stakeholder; (2) developing an initial synthesis of the findings of the eligible studies; (3) exploring relationships within and between studies; and (4) assessing the robustness of the synthesized evidence [82]. These steps do not have to be conducted exactly according to the order of this framework and will be conducted iteratively by the three topic experts (RMR, LR, and LL) [82]. Disagreements will be resolved through discussions and persistent disagreements will be resolved through the arbitrage of a fourth author (NDG) or through contacting pertinent authors. Each phase of this synthesis will be presented in the results section of the systematic review [82]. Each step of this four phase framework for a systematic narrative synthesis is presented under here:

- Phase 1. Developing a theory why and how each barrier or facilitator could affect the implementation of OMIs for each linked stakeholder
  Theories of how barriers or facilitators could affect the implementation of OMIs for various stakeholders will be initially developed during the discussions on ‘potential’ barriers and facilitators to the implementation of OMIs in clinical practice prior to the selection and data collection process (see ‘Data collection process’ section). These theories will be fine-tuned after the data extraction procedures. We expect that most identified barriers and facilitators are obvious implementation constructs for the pertinent stakeholders and do not require much additional theory to why and how they could affect the implementation of OMIs. However, such causal links could be more difficult to explain for certain ‘unexpected’ barriers and facilitators. In those circumstances, we will present ‘hypothetical’ associations descriptively or in a diagram or both [82].
- Phase 2. Developing an initial synthesis of the findings of the eligible studies
  Our tables with the characteristics and findings of included studies will be consulted for this preliminary synthesis. We will also revisit the data collection tables and search for additional items that might have been overlooked and could influence this synthesis (Additional files 2 and 3). To facilitate the synthesizing process, we will reorganize data items, fine-tune textual descriptions of each included study, assess the validity of outcome measures, and create new tables with implementation constructs and pertinent stakeholders to improve visualization of the data [82]. We will assess whether barriers and facilitator can be linked to one of the five domains defined by [25], i.e., intervention characteristics, outer setting, inner setting, characteristics of the individuals involved, and the process of implementation. The prevalence statistics of our primary outcomes will be subsequently consulted and theories about the direction and magnitude of the effects of specific implementation constructs on pertinent stakeholders will be developed.
- Phase 3. Exploring relationships within and between studies
  In this phase, we will assess whether and how variables within and between studies could have influenced outcomes. For this purpose, we will (1) explore the characteristics and findings of each selected study and compare them with each other; (2) consult our tables with ‘a priori’ defined sources of heterogeneity (Additional file 5) and will consider sources of diversity that are identified ‘post hoc’ [65, 70]. Various tools will be used to further explore relationships within and between studies: (1) investigating statistical heterogeneity (Additional file 5); (2) visualizing of statistical heterogeneity in forest plots [68]; (3) conducting subgroup analyses and assessing moderator variables (see ‘Subgroup analyses and meta-regression’ section) [82]. Based on these assessments, we will modify and fine-tune our initial synthesis and will explain the rationale for these changes.
- Phase 4. Assessing the robustness of the synthesized evidence
  The robustness of the synthesized evidence will depend on (1) the number and size of the eligible studies; (2) within and between study diversity; (3) risk of bias assessments (magnitude and direction); (4) the consistency of the outcomes between studies; (5) the magnitude of the outcomes; and (6) the presence of publication bias. To assess the robustness of this evidence, we will (1) weigh the role of these variables; (2) revisit the data collection forms and the critical appraisal tools to assess whether items have been overlooked; and (3) contact authors to obtain additional information.

### Meta-biases and confidence in the cumulative evidence

Meta-bias refers to the biased selection of research data and covers both reporting bias (selective outcome reporting) and publication bias [43]. We will apply the following strategies for dealing with reporting bias:

1. We will assess whether the protocol of a pertinent research study was published prior to the recruitment of patients [43]. Literature searches and screening of the Clinical Trial Register at the International Clinical Trials Registry Platform of the World Health Organization will be conducted for this purpose [85].
2. When protocols will be identified, we will assess discrepancies between the outcomes planned in the protocol and those reported in the final manuscript. When no protocol will be found, we will scrutinize the eligible article for unclear or missing outcomes.
3. If necessary, research protocols or additional information on the outcomes will be requested from the authors of the research study. We will also compare overall outcomes with and without including the data obtained from contacted authors.
4. Subsequent strategies for ‘dealing with missing data for a quantitative synthesis’ were described under this heading.

We will contain the risk of publication bias through (1) our broad spectrum search strategy and extensive searches of the literature that also include the gray literature; (2) our protocol for assessing multiple publications; (3) searching studies and collecting data by three experienced operators; and (4) contacting pertinent stakeholders that could provide information on unpublished or ongoing studies. We will apply a combination of four methods in our quantitative synthesis to assess the presence and the possible impact of publication bias [86]. These methods are presented under here and are all based on the assumption that publication bias increases as study sample sizes decrease [86]:

1. Plot the studies according to their sample size in a forest plot and assess a possible relationship between the sample size and the effect size.
2. Conduct subgroup analyses in which articles published in peer-reviewed journals are compared with those published in the gray literature and assess whether these latter publications tend to have smaller effect sizes.
3. Compare the summary outcomes of the random versus the fixed effects model to assess the role of small sample sizes on these effect measures.
4. Explore the relationship between study size and effect size by displaying these values in funnel plots and assessing their symmetry [50]. We will also assess the best estimate of the unbiased effect size using the Duval and Tweedie’s Trim and Fill procedure [87]. However, these graphs will only be designed when ten or more studies are eligible [50, 72].

For the assessment of the strength of the body of evidence, we will consult the guidelines described by the GRADE approach [88] and will weigh the variables described under the subheading ‘Assessing the robustness of the synthesized evidence’ in the ‘Qualitative synthesis’ section. We will not score the ‘levels of evidence’ according to the GRADE approach. Our research questions do not qualify for this approach, because they do not address questions about interventions, management strategies, or policies [89].

### Differences between the protocol and the review

All preferred reporting items for systematic review and meta-analysis protocols (PRISMA-P) 2015 and the pertinent pages are listed in additional file 6 [42, 43]. We will fully report all changes in the methods during the conduct of this research study compared with those planned in the protocol. We will describe the type, timing, and the rationale of each of these modifications. We will also report the consequences of these changes on the direction, the magnitude, and the validity of the outcomes [90].

## Discussion

Orthodontic research focuses primarily on the effectiveness of new health technologies and little information is published on their subsequent implementation in clinical practice. Scoping searches showed that this is the first protocol for a systematic review that assesses barriers and facilitators to the implementation of a new orthodontic technique.

The strengths of this systematic review include the following: (1) it will focus on a broad spectrum of stakeholders and interventional procedures. This is important because our findings could demonstrate that not patient-related variables but doctor-or office staff-related constructs are the key barriers to the implementation of OMIs. Broad eligibility criteria could also reveal unexpected barriers or facilitators; (2) research procedures are reported in great detail in this protocol, which will improve transparency and will facilitate the update of this systematic review by future reviewers; (3) extensive literature searches with broad spectrum search strategies will be undertaken, which will accept low precision and will aim at high sensitivity; (4) literature searches and data extraction will be conducted independently by three topic experts; and (5) this research study will be conducted by experienced reviewers and methodologists, who have published various systematic reviews on OMIs [38–41].

A weakness of this systematic review could be that some answers to our research questions will not be addressed or remain suboptimal, because studies on implementation issues are of low quality or are deficient in the orthodontic literature. However, the identification of such knowledge gaps could be a stimulus for a new wave of research studies that will assess why certain stakeholders refrain from using specific orthodontic therapies and which strategies can be applied to deal with these issues.

Patients, clinicians, staff members, researchers, guideline developers, policy makers, and orthodontic companies will all be beneficiaries of the outcomes of this systematic review. Its findings could also apply to a variety of interventional procedures in dentistry and oral surgery. Redirecting research on OMIs towards studies that address implementation issues could ultimately reduce research waste and improve patient health experiences [91–93].

## Additional files

Additional file 1:
**Protocol for contacting authors.** (DOCX 27 kb) Additional file 2:
**Data collection forms.** (DOCX 28 kb) Additional file 3:
**Characteristics and findings of included studies.** (DOCX 22 kb) Additional file 4:
**Critical appraisal tools.** (DOCX 17 kb) Additional file 5:
**Heterogeneity. Sources of heterogeneity and strategies for dealing with heterogeneity.** (DOCX 21 kb) Additional file 6:
**PRISMA-2015 Checklist.** (DOCX 25 kb)

## Abbreviation

OMIs: Orthodontic mini implants
