# Supplementary material for: Barriers and facilitators to the implementation of orthodontic mini-implants in clinical practice: a protocol for a systematic review and meta-analysis
Source: Syst Rev. 2016 Feb 5;5:22. doi: 10.1186/s13643-016-0198-4 (PMC4743120; doi:10.1186/s13643-016-0198-4)
Supplement: Additional file 1: — Protocol for contacting authors. (DOCX 27 kb) [file 13643_2016_198_MOESM1_ESM.docx]

**Additional file 1.**

**Protocol for contacting authors**

Our protocol for contacting authors is based on protocols that we used in previous systematic reviews on OMIs [38-41]. The objectives of this protocol are to obtain additional information on: 1) the eligibility of specific research studies; and 2) unclear or missing data in primary research studies. Our protocol is presented as a step by step process. All emails sent to the contacted authors will be accompanied by the research protocol of our systematic review and all contacting attempts are conducted at 2 week intervals.

**Step 1.** In our initial email we will ask reference authors whether they are willing to provide pertinent additional information on their research studies. Authors are only asked to answer this question with a ‘Yes’ or a ‘No’. We decided to use this simple ‘Willingness to reply’ format, because: 1) it can be used to confirm the validity of the email address of a reference author; 2) researchers might initially be hesitant to reply when confronted with large numbers of questions on their research study. An example of our initial email is presented in exemplary email 1.

**Step 2.** When no response is received after 2 weeks, a reminder email will be send to the reference author.

**Step 3.** When after another 2 weeks the reference author has still not responded, the initial email will be send to at least one of the co-authors.

**Step 4.** In the case of no response, a reminder of this email will be sent after 2 weeks.

**Step 5.** When after another 2 weeks still no response has been obtained, steps 1-4 will be repeated from another email address. This method was chosen, because our email address could be classified as a ‘spam’ email address by the internet provider of the contacted authors and therefore become invisible to these authors [38].

**Step 6.** Two weeks after the last email, we will send an email (Exemplary email 2) with pertinent questions to all authors that responded our ‘Willingness to reply’ question with a ‘Yes’. Questions will be presented as a combination of open-and closed-ended questions. For each specific research design we will consult the pertinent checklists of the Equator Network to fine-tune these questions [33]. We aim at not exceeding a total of 10 questions.

**Step 7.** We will summarize the proceedings of our methods for contacting of authors in separate exemplary tables (Tables 1 and 2) of this additional file. In the final systematic review we will present a table that summarizes these proceedings and also lists the consequences of this information on e.g. eligibility, primary and secondary outcomes, and the assessment of risk of bias. We will report this information separately from the data extracted from the original research studies and do not incorporate these data in our analysis, because the validity of data obtained through contacted authors is currently unknown [59].

**Exemplary email 1. Assessment of author’s willingness to reply to questions of systematic reviewers** [40]

| Dear Professor ………….,  We are currently conducting a systematic review* on orthodontic mini implants and would like to obtain some additional information on your article:    “ …………………………………………………………….”  In the near future we would like to ask you some simple questions on this publication. Answering these questions will probably not require more than 2 minutes of your time.  In this email we are only interested to find out whether you are willing to provide us with this additional information or not.  We therefore ask you the following question:  **Are you in the near future willing to respond to some simple questions pertinent to the indicated article ?**  - If you are willing to respond to our questions please reply to this email, a "YES" is enough.    - If you are not willing to respond to our questions please respond this email with a “NO”. In case, you do not reply, we will send you a reminder email 14 days after the current email.  -In case you respond with a “NO” or do not reply to this email and to the reminder, we will not further contact you, but will contact at least one of the other co-authors of your research study.  Thank you very much for your cooperation.  Sincerely,  Reint Meursinge Reynders and Nicola Di Girolamo  ----------------------------------------------------- *This systematic review originated as an assignment for the Evidence Based Health Care program at the University of Oxford, UK. The aforementioned individuals are subjectively responsible for the information requested. |
| --- |

**Exemplary email 2. Research questions for authors to obtain additional data on their research studies***[40]

| Dear Professor ………..,  In an earlier mail we asked you to answer some simple questions on your research article listed under here.  Your answers could be a great contribution to improve on the quality of our systematic review**. The published protocol of our systematic review can be found in the attachment.  As we wrote in our previous email we would like to ask you some (9 in total) short questions regarding your article:  “ …………………………………………………………….”  All questions are combined closed-and open-ended questions.  If you answer a question with a YES, we would like you to give a short description.  Definitions of specific items are presented under the Question and Answer table.   \| **Questions** \| **Answer** \| \| --- \| --- \| \| ***Was random sampling^1^ of a particular subset of the population applied?***  If you answer with a **YES**, could you please describe how this procedure was conducted  If you answer with a **NO**, it is not necessary to provide any additional information \|  \| \| ***Were particular groups of persons excluded?***  If you answer with a **YES**, could you please describe which participants were excluded and why  If you answer with a **NO**, it is not necessary to provide any additional information \|  \| \| ***Were consecutively treated^2^ participants enrolled?***  If you answer with a **YES**, could you please describe how this procedure was conducted  If you answer with a **NO**, it is not necessary to provide any additional information \|  \| \| ***Was a power calculation conducted to determine the adequate sample size?***  If you answer with a **YES**, could you please describe how this procedure was conducted and the outcome of this calculation  If you answer with a **NO**, it is not necessary to provide any additional information \|  \| \| ***Did all stakeholders refer to the same interventional procedure?***  If you answer with a **YES**, could you please describe the interventional procedure  If you answer with a **NO**, it is not necessary to provide any additional information \|  \| \| ***Was the setting^3^ the same for all stakeholders ?***  If you answer with a **YES**, could you please describe the setting  If you answer with a **NO**, it is not necessary to provide any additional information \|  \| \| ***Were all stakeholders interviewed by the same interviewer?***  If you answer with a **YES**, could you please describe who interviewed all stakeholders and his/her experience with interviewing  If you answer with a **NO**, it is not necessary to provide any additional information \|  \| \| ***Was an interview guide used for conducting the interview?***  If you answer with a **YES**, could you please describe whether questions were open-ended, closed-ended or a mix of these questions  If you answer with a **NO**, it is not necessary to provide any additional information \|  \| \| ***Did you record the response rate of the surveyed population?***  If you answer with a **YES**, could you please provide the response rate  If you answer with a **NO**, it is not necessary to provide any additional information \|  \|   **Definitions of the various items:**  **^1^Random sample**  The “Glossary of terms in the Cochrane Collaboration” [60] defines a random sample as ‘A group of people selected for a study that is representative of the population of interest. This means that everyone in the population has an equal chance of being approached to participate in the survey, and the process is meant to ensure that a sample is as representative of the population as possible’.  **^2^Consecutively treated participants**  Refers to the inclusion of all participants treated with the interventional procedure over a period of time.  **^3^Setting**  The setting refers to the location where the research study was conducted, e.g., private practice, university setting, hospital etc.  --------------------------------------------------------------------------------------------------------------------------------  We thank you again for your help and time.  We will send you a copy of our systematic review as soon as it will be accepted for publication.  In the case that you have changed your mind and are not willing to reply to these questions, could you please explain the rationale for this decision.  Thank you again very much for your cooperation.  Sincerely,  Reint Meursinge Reynders and Nicola Di Girolamo  **This systematic review originated as an assignment for the Evidence Based Health Care program at the University of Oxford, UK.  The aforementioned individuals are subjectively responsible for the information requested. |
| --- | --- | --- | --- | --- | --- | --- | --- | --- | --- | --- | --- | --- | --- | --- | --- | --- | --- | --- | --- | --- |

**Table 1. Exemplary outcomes of (2 fictive) authors’s willingness to reply to questions of systematic reviewers**

| **^1^Article and year** | **^2^Contacted author(s)** | **^3^Number of contacting attempts and ^4^number of days to get a reply** | **^5^Reminder mails?** | **^6^Co-authors contacted?** | **^7^Willingness to reply?** |
| --- | --- | --- | --- | --- | --- |
| Author A (2014) | Author A | 1 attempt  Response same day | No | No | Yes |
| Author B  (2015) | Author B and co-author C | 4 attempts  Response after 80 days | Yes | Yes | No |

**^1^Article and year:** Presentation of the first author and the year of publication

**^2^Contacted author(s):** Presentation of each author that was contacted, i.e., the reference author and co-authors

**^3^Number of contacting attempts:** The number of contacting attempts refers to:

- The total number of attempts by email to get a response from a contacted author with either a ‘Yes’ or a ‘No’ on his/her willingness to reply to questions on his/her research studies. This number includes the total number of attempts of contacting both the reference author and potential co-authors.
- The initial email or the subsequent reminder email are each counted as 1 attempt. Emails that are reported as ‘failed’ by the internet provider, because they did not arrive at the desired email address are not counted as attempts.
- Our emails could also be identified as ‘spam mail’ by the receiving internet provider and we will therefore also send emails from our secondary email address (See step 5). When authors reply to this secondary email address, we will not count the earlier email attempts from our primary email address.
- As soon as authors have replied with either a ‘Yes’ or a ‘No’, we will not consider any successive email correspondence as part of the attempt count.
- Ideally only 1 contacting attempt is made by the systematic reviewers.

**^4^Number of days to get a reply:** The number of days to get a reply refers to:

- The total number of days necessary to get a response from a contacted author with either a ‘Yes’ or a ‘No’ on his/her willingness to reply to questions on his/her research studies. This number of days includes the total number of days of contacting both the reference author and potential co-authors to get such a reply.
- The day of sending the initial email to the reference author is the starting point for recording the ‘Number of days to get a reply’. The day of receiving a ‘Yes’ or a ‘No’ to our question from either the reference author or a co-author is the final recording of this outcome.
- We do not count days when emails are reported as ‘failed’ by the internet provider, because these letters did not arrive at the desired email address. When authors do not respond to our primary email address, but only to our secondary email address, we will only record the days necessary to get a reply to our email from the secondary address.
- As soon as authors have replied with either a ‘Yes’ or a ‘No’, we will not count the days of any successive email correspondence.
- Ideally authors respond the same day to the initial ‘Willingness to reply’ email.

**^5^Reminder mails:** Score with a ‘Yes’ or a ‘No’

**^6^Co-authors contacted:** Score with a ‘Yes’ or a ‘No’

**^7^Willingness to reply:** Score with a ‘Yes’ or a ‘No’

**Table 2. Exemplary outcomes of answering by (2 fictive) contacted authors to research questions of systematic reviewers**

| **^1^Article and year** | **^2^Contacted author(s)** | **^3^Number of contacting attempts and ^4^number of days to get a reply to our research questions** | **^5^Reminder mails?** | **^6^Prevalence of research questions answered** |
| --- | --- | --- | --- | --- |
| Author A (2014) | Author A | 1 attempt | No | 9 of 9 questions |
| Author B  (2015) | Author B and co-author C | 5 attempts  Response after 80 days | Yes | 2 of 6 questions |

**^1^Article and year:** Presentation of the first author and the year of publication

**^2^Contacted author(s):** Presentation of each author that was contacted, i.e., the reference author and co-authors

**^3^Number of contacting attempts:** The number of contacting attempts refers to:

- The total number of attempts to get a reply to our email with research questions. These emails will only be sent to authors that answered our ‘Willingness to reply email’ with a ‘Yes’.
- Eligible replies vary from ‘unwillingness to answer’ our research questions to answering some or all of them. Subsequent emails to get explanations or to obtain data on unanswered questions are not counted as additional attempts.
- Ideally only 1 contacting attempt is made to get a reply to our email with research questions.

**^4^Number of days to get a reply:** The number of days to get a reply refers to:

- The total number of days necessary to get an eligible reply from a contacted author.
- The day of sending the email with research questions to a pertinent author is the starting point for recording the ‘Number of days to get a reply’. The day of receiving an eligible outcome is the final recording of this outcome
- Ideally authors respond to all questions on the same day that they receive our email with research questions.

**^5^Reminder mails:** Score with a ‘Yes’ or a ‘No’

**^6^Prevalence of research questions answered:** Score the prevalence, i.e. the number of research questions answered out of total number of questions asked
