# Supplementary material for: Barriers and facilitators to the implementation of orthodontic mini-implants in clinical practice: a protocol for a systematic review and meta-analysis
Source: Syst Rev. 2016 Feb 5;5:22. doi: 10.1186/s13643-016-0198-4 (PMC4743120; doi:10.1186/s13643-016-0198-4)
Supplement: Additional file 2: — Data collection forms. (DOCX 28 kb) [file 13643_2016_198_MOESM2_ESM.docx]

**Additional file 2. Data collection forms**

The pilot-tested data collection forms with a description of each extracted item are presented in the tables under here. Pertinent items to facilitate the assessment of risk of bias are also included.

**Data collection form 1. Source and eligibility**

| **Entry** | **Description and character of the information** |
| --- | --- |
| Name of reviewer | Report the name of the reviewer. |
| Authors, title, journal of article | List authors, title, and journal. |
| Source of article | Describe how the article was retrieved and from which database, e.g. Medline, grey literature, hand searching of review articles etc. |
| Language of the article | Describe in which language the article was published. |
| Registration of study | Describe if study was registered, under what number, and in which register. |
| Duplicate publication | Describe whether the study as a whole or parts of the study are suspect of having been published more than once.  If suspicion of duplicate publication has been identified, the reviewer should also present the reference of pertinent duplicate studies and which parts of the study overlap. |
| Eligible/not eligible | Confirm eligibility.  If not eligible, explain the reason for exclusion. |
| Purpose | Copy the objectives of the paper according to the authors. |

**Data collection form 2. Miscellaneous ***

| **Entry** | **Description and character of the information** |
| --- | --- |
| Funding source | Describe the funding source and indicate if it is an implant company. |
| Key conclusions of study authors | Describe the main conclusions by the authors of the research study. |
| Important comments by the study authors | Describe important comments by the authors of the research study. |
| References to other relevant studies | List references of other relevant studies that should be retrieved. |
| Key conclusions of the review authors | Describe the main conclusions by the review authors. |
| Major strength according to review authors | Describe the major points of strength of the research study according to the review authors. |
| Major weaknesses according to review authors | Describe the major points of weakness of the research study according to the review authors. |
| Contacting authors | Indicate whether authors should be contacted and indicate what additional information is necessary. |
| Discuss with statistician | Describe points that need to be discussed with a statistician. |
| Points of interest | Describe points of interest, e.g. an unexpected barrier or facilitator to the implementation of OMIs |
| Future studies | Describe ideas for future studies suggested by the authors or reviewers. |
| Other comments by review authors | Any additional comment that has not been covered. |

*Locate where information on each item can be found, e.g., Page 12 column 3.

**Data collection form 3. ‘Study design and selection procedures’ ***

| **Entry** | **Description and character of the information** |
| --- | --- |
| Date of the study | Describe when the study was started and completed. |
| Study design | Was the study design reported, e.g., survey, questionnaire, interview, focus group etc.?  If answered with ‘Yes’, describe the study design.  If answered with ‘No’, describe the study design. |
| Selection procedures general | Were the selection procedures of the stakeholders, e.g., random sampling, consecutively treated, case control design etc. reported?  If answered with ‘Yes’, describe the selection procedures. |
| Consecutively treated | Did the authors report on consecutive treatment of stakeholders?  If answered with ‘Yes’, describe this entry. |
| Sequence generation | Did the authors report on sequence generation, e.g., random or non random?  If answered with ‘Yes’, describe the sequence generation.  1) Random sequence generation ? Describe the type of randomization, e.g. computerized.  2) Non-random sequence generation ? |
| Allocation concealment | Did the authors report on concealment of allocation?  If answered with ‘Yes’, describe blinding of pertinent operators and stakeholders, e.g., patients, clinicians, test operators, and personnel during the allocation procedures. |
| Case-control design | Was a case-control design used?  If answered with ‘Yes’, describe how stakeholders were enrolled according to a case-control design. |
| Power calculation | Did the authors conduct a power calculation?  If answered with ‘Yes’, describe how the power calculation was conducted. |
| Inappropriate exclusions | Were single stakeholders or specific groups of stakeholders inappropriately excluded during any phase of the selection process?  If answered with ‘Yes’, describe these procedures |
| Approved by ethical board | Was the research study approved by an ethical board?  If answered with ‘Yes’, describe this approval and the components of the ethical board. |
| Risk of bias | Could any of the entries described in this table have caused biased study outcomes?  If answered with ‘Yes’, present the rationale for this judgment.  Present the magnitude of the bias (High, Low, Unclear) for each entry that was scored with a Yes. Also indicate the direction of this bias on the outcomes, i.e. increase or decrease of the magnitude of outcomes |

*Locate where information on each item can be found, e.g., Page 10 column 2.

**Data collection form 4. ‘Stakeholders’ ***

| **Entry** | **Description and character of the information** |
| --- | --- |
| Type of stakeholder | Was the type of stakeholder, i.e., patients, clinicians, office staff, clinic owners, policy makers etc. clearly reported?  If answered with ‘Yes’, describe the type of stakeholder |
| Type of ‘patient’ stakeholders | Describe this type of stakeholder, e.g., patients that finished treatment with palatal OMIs less than 3 months previously etc. |
| Type of ‘clinician’ stakeholders | Describe this type of stakeholder, e.g. members of a dental association etc. |
| Type of ‘clinic staff’ | Describe this type of stakeholder, e.g. secretaries, assistants, laboratory staff etc. |
| Type of ‘policy makers’ | Describe this type of stakeholder, e.g., Governmental body, organization of professionals etc. |
| Other types of stakeholders | Describe other pertinent stakeholders, e.g. family members etc. |
| Number, sex, and age of stakeholders (including subgroups) | e.g. 20 males and 20 females mean age 23.6 ± 8.1 years  Indicate particular subgroups if indicated, e.g., experienced versus non-experienced surgeons or patients that had undergone the intervention and those that have not, differences in interventions etc. |
| Ethnicity | Was the ethnicity of the stakeholders reported?  If answered with ‘Yes’, describe the ethnic group(s) and if applicable, present the number of stakeholders per ethnic subgroup. |
| Socio-economic status | Was the socio-economic status of the stakeholder(s) described?  If answered with ‘Yes’, describe the socio-economic status and if applicable, present the number of stakeholder(s) per socio-economic subgroup. |
| Experience with the interventional procedure | Was the experience of the stakeholders with the interventional procedure reported?  If answered with ‘Yes’, describe the experience of stakeholders with this procedure, e.g., for patients (had they undergone the procedure or not), for clinicians (had they conducted the interventional procedure or not), etc. Include some additional variables that could be pertinent to this domain and if applicable, present the number of stakeholders per subgroup. |
| Co-existent conditions | Were co-existent conditions described?  If answered with ‘Yes’, describe co-existent conditions for example:   1. For patients this could be possible past interventions or co-interventions etc. Assess whether these interventions could have influenced the outcomes. 2. For clinicians and policy makers, prejudiced views on the interventional procedures etc.   If applicable, present the number of stakeholders per subgroup. |
| Medical and dental health condition | Were medical variables, e.g. uncontrolled diabetes, osteoporosis, smoking, pharmacological treatment etc. reported?  If answered with ‘Yes’, present the type of medical variables and the number of stakeholders per subgroup.  Were dental variables, e.g. periodontal disease, loss of teeth etc. reported?  If answered with ‘Yes’, present the type of dental variables and if applicable, present the number of stakeholders per subgroup. |
| Other stakeholder-related variables | Were other stakeholder-related variables described that could have influenced outcomes?  If answered with ‘Yes’, describe these ‘other’ stakeholder-related variables and how they could have influenced outcomes. |
| Risk of bias | Could any of the entries described in this table have caused biased study outcomes?  If answered with ‘Yes’, present the rationale for this judgment.  Present the magnitude of the bias (High, Low, Unclear) for each entry that was scored with a Yes. Also indicate the direction of this bias on the outcomes, i.e. increase or decrease of the magnitude of outcomes |

*Locate where information on each item can be found, e.g., Page 10 column 2.

**Data collection form 5. ‘Setting, country and city’ ***

| Setting | Was the setting, e.g. private practice, university clinic etc., reported?  If answered with ‘Yes’, describe the setting of the research study and  if applicable, present the number of stakeholders per subgroup. |
| --- | --- |
| Country/city | Was the country(ies) and city(ies) or other geographical contexts (outside city) where the research study was conducted described?  If answered with ‘Yes’, describe in which country(ies), city (or not) the research study was conducted and if applicable, present the number of stakeholders per country or city subgroup. |
| Other setting-related variables | Were other setting-related variables described that could have influenced outcomes?  If answered with ‘Yes’, describe these ‘other’ setting-related variables and how they could have influenced outcomes. |
| Risk of bias | Could any of the entries described in this table have caused biased study outcomes?  If answered with ‘Yes’, present the rationale for this judgment.  Present the magnitude of the bias (High, Low, Unclear) for each entry that was scored with a Yes. Also indicate the direction of this bias on the outcomes, i.e. increase or decrease of the magnitude of outcomes |

*Locate where information on each item can be found, e.g., Page 10 column 2.

**Data collection form 6. ‘Interventions’***

| **Entry** | **Description and character of the information** |
| --- | --- |
| Definition of the interventional procedure | Did the authors define the interventional procedure(s) for which barriers and facilitators were identified?  If answered with ‘Yes’ copy the definition of the interventional procedure according to the authors. |
| Specified or non specified interventions | Did the authors refer to a ‘specified’ or ‘non specified intervention’ ?  ***Specified interventions:*** these interventions refer to a specific phase or type of the interventional procedure. Phases of the intervention refer to the: anesthetics, implant insertion, orthodontic treatment with OMIs, implant removal, or the healing phase. Types of interventions refer to the: implant type and dimensions, number of implants, use of plates, the surgical procedure, implant location, timing and forces of orthodontic loading etc. [41].  ***‘Non specified’ interventions:*** these interventions refer to “any orthodontic treatment with OMIs”. Additional information on the specific phase or type of the interventional procedure is not provided  If answered with ‘Yes’ describe the type of interventional procedure. |
| Plates ? | Were plates connected to the OMIs?  Describe if answered with ‘Yes’. |
| Type and number of OMIs | Were the type and number of OMIs presented?  Describe if answered with ‘Yes’, e.g., 2 Quattro*implants diameter 1.5 mm and length 9 mm (*PSM Medical Solutions; Tuttlingen, Germany) |
| Location ? | Was the location of the insertion site described?  Describe if answered with ‘Yes’, e.g., palate |
| Duration of the intervention | Was the duration of the intervention described ?  Describe the duration of the intervention (in weeks) if answered with ‘Yes’. |
| Additional specification of the interventional procedure | Did the authors present any additional information on the interventional procedure?  If Yes, specify. Examples of such specifications are presented under here:   1. With or without plates 2. Number of implants per patient 3. Implant type (code name and number) and the name of the company 4. Implant form, i.e., tapered, cylindrical or a combination of these forms 5. Implant dimensions, i.e., diameter and length 6. Drilling design, i.e., self-drilling or a pre-drilling design 7. Insertion technique, i.e., self-drilling or pre-drilling technique 8. Flap or flapless surgical intervention 9. Location of implant insertion, i.e., vestibular or palatal in addition to the location between specific teeth 10. Location with respect to type of mucosa, i.e., in the keratinized or non-keratinized mucosa 11. Exposure of implant, i.e., exposed or non-exposed under the mucosa 12. Orthodontic loading, i.e., immediately after implant insertion or after a certain loading period 13. Implant removal with or without anesthetics etc. 14. Adverse effects, i.e., pain and discomfort, inflammation of oral tissues, and biologic damage [41] |
| Interventional subgroups | Were subgroups for interventional procedures presented?  Define subgroups if answered with ‘Yes’. |
| Other intervention related variables | Were other interventional related variables described that could have influenced outcomes?  If answered with ‘Yes’, describe these ‘other’ intervention related variables and how they could have influenced outcomes. |
| Risk of bias | Could any of the entries described in this table have caused biased study outcomes?  If answered with ‘Yes’, present the rationale for this judgment.  Present the magnitude of the bias (High, Low, Unclear) for each entry that was scored with a Yes. Also indicate the direction of this bias on the outcomes, i.e. increase or decrease of the magnitude of outcomes |

*Locate where information on each item can be found, e.g., Page 10 column 2.

**Data collection form 7. ‘Outcomes’***

| **Entry** | **Description and character of the information** |
| --- | --- |
| Type of barrier or facilitator to the implementation of OMIs | List barriers or facilitators to the implementation of OMIs that were identified in the research study. |
| Definitions of barriers or facilitators to the implementation of OMIs | Did the author(s) present definition(s) of the barrier or facilitator to the implementation of OMIs?  If answered with ‘Yes’, copy the definition of these items according to those presented by the author(s) of the selected study. |
| Clarity of definitions of barrier or facilitator to the implementation of OMIs | Was the definition of the barrier or facilitator to the implementation of OMIs sufficiently clear, in the sense that there was no doubt to which item the authors were referring?  If not explain. |
| Time point(s) of scoring outcomes | Were time point(s) of identifying barriers and facilitators to the implementation of OMIs scored ?  Score time point(s) (in days) if answered with ‘Yes’, i.e., (1) pre-intervention recordings, i.e., recordings prior to the interventional procedure (2) immediate post-intervention recordings, i.e., recordings within 2 weeks after the completion of the interventional procedure; and (3) long-term post-intervention recordings, i.e., recordings after more than 2 weeks after the completion of the interventional procedure ? |
| Multiple time points for recording outcomes | Were outcomes recorded at more than 1 time point, e.g., pre-and post intervention outcomes?  Describe the multiple time points for measuring outcomes and their time intervals (in days) if answered with ‘Yes’.  Describe whether this interval was considered correct, too short or too long. Explain the rationale for this judgment. |
| Response rate of stakeholders that were contacted to participate in surveys | Was the response rate of the stakeholders reported?  If answered with ‘Yes’, present the prevalence of the contacted stakeholders that actually answered the survey, i.e., The number of stakeholders that answered the survey questions/The total number of stakeholders that was contacted to answer these questions  Present the response rate for example as: 200/1000 |
| Inclusion in analysis  (Lost to follow-up) | Were all surveyed/interviewed stakeholders also included in the data analysis.  If answered with ‘No’, present total numbers of lost to follow-up.  If answered with ‘No’, Present the number of stakeholders that were lost to follow-up/The total number of stakeholders that answered the survey or participated in the interviews. Present this statistic as the lost to follow-up rate for example as 200/1000  Present explanations for not being included in the analysis. |
| Prevalence of pertinent barriers and facilitators to the implementation of OMIs | Was the prevalence of identified barriers and facilitators among the surveyed or interviewed pertinent stakeholders reported ?  If answered with ‘Yes’, this statistic is calculated as:  ***Prevalence of an identified barrier or facilitator*** =  *The number of stakeholders that scored a particular construct as a barrier or facilitator to the implementation of OMIs in clinical practice/*  *The total number of stakeholders that scored on the role of this particular construct as a barrier or facilitator to the implementation of OMIs in clinical practice*  This prevalence will be presented for example as: 30/50 |
| Quality of the outcome assessors | Was the quality, experience, and competence of the outcome assessor(s) described ?  If answered with ‘Yes’, describe these characteristics |
| Number of outcome assessors | Was the number of outcome assessors presented?  If answered with ‘Yes’, present the number of outcome assessors. Ideally more than one operator assesses the outcomes in order to avoid inadequate outcome assessments or exclusions of outcomes. |
| Calibration of outcome assesors | Were outcome assessors calibrated?  If answered with ‘Yes’, describe how outcome assessors were calibrated. |
| Intra-or inter-operator differences | Were intra-or inter operator differences of outcome assessors presented?  If answered with ‘Yes’, describe these differences.  Assess whether these differences are small enough to be ignored. |
| Other procedural variables | Could other procedural variables have influenced outcomes?  If answered with ‘Yes’, describe these procedural variables and how they could have influenced outcomes. |
| Risk of bias | Could any of the entries described in this table have caused biased study outcomes?  If answered with ‘Yes’, present the rationale for this judgment.  Present the magnitude of the bias (High, Low, Unclear) for each entry that was scored with a Yes. Also indicate the direction of this bias on the outcomes, i.e. increase or decrease of the magnitude of outcomes |
| **Secondary outcome**  Prevalence of clinicians that do not use OMIs | Was the prevalence of clinicians that do not use OMIs reported?  If answered with ‘Yes’, this statistic is calculated as:  ***The prevalence of clinicians that do not use OMIs*** *=*  *The number of clinicians that do not use OMIs/*  *The total number of surveyed clinicians that reported on the use of OMIs in clinical practice*  Present the prevalence for example as: 200/1000 |
| Other pertinent outcomes on the use of OMIs | Were other pertinent outcomes on the use of OMIs by clinicians reported?  If answered with ‘Yes’ present such outcomes, e.g., the number of implants placed per clinician per year. |

*Locate where information on each item can be found, e.g., Page 10 column 2.

**Collection form 8. ‘Flow and timing’***

| **Entry** | **Description and character of the information** |
| --- | --- |
| Flow diagram | Design a diagram of the flow of the stakeholders and the timing of the various research phases from the start of the selection procedures to the completion of the recording of outcomes. This flow diagram should be divided in 5 research phases: 1) the population from which stakeholders were selected; 2) selection procedures; 3) conducting the research and data collection; 4) data analysis; and 5) reporting data. During each of these phases the number of stakeholders will be presented as well as the duration and division (include time intervals) of individual research phases. When possible a rationale for withdrawals and missing data should be given. |
| Other flow and timing related variables | Could other flow and timing related variables have influenced outcomes?  If answered with ‘Yes’, describe these ‘other’ flow and timing related variables and how they could have influenced outcomes. |
| Risk of bias | Could any of the entries described in this table have caused biased study outcomes?  If answered with ‘Yes’, present the rationale for this judgment.  Present the magnitude of the bias (High, Low, Unclear) for each entry that was scored with a Yes. Also indicate the direction of this bias on the outcomes, i.e. increase or decrease of the magnitude of outcomes |

*Locate where information on each item can be found, e.g., Page 12 column 3.

**Data collection form 9. ‘Funding’***

| **Entry** | **Description and character of the information** |
| --- | --- |
| Funding sources | Was the funding of the research study reported?  If answered with ‘Yes’, describe the funding sources of the research study. |
| Conflict of interest | Could the authors or any of the stakeholders have a conflict of interest?  If answered with ‘Yes’, describe the type of conflict of interest and the pertinent stakeholders. |
| Risk of bias | Could any of the entries described in this table have caused biased study outcomes?  If answered with ‘Yes’, present the rationale for this judgment.  Present the magnitude of the bias (High, Low, Unclear) for each entry that was scored with a Yes. Also indicate the direction of this bias on the outcomes, i.e. increase or decrease of the magnitude of outcomes |

*Locate where information on each item can be found, e.g., Page 10 column 2.
