# Supplementary material for: Barriers and facilitators to the implementation of orthodontic mini-implants in clinical practice: a protocol for a systematic review and meta-analysis
Source: Syst Rev. 2016 Feb 5;5:22. doi: 10.1186/s13643-016-0198-4 (PMC4743120; doi:10.1186/s13643-016-0198-4)
Supplement: Additional file 3: — Characteristics and findings of included studies. (DOCX 22 kb) [file 13643_2016_198_MOESM3_ESM.docx]

**Additional file 3.**

**Characteristics and findings of included studies**

The procedures to extract and categorize primary outcomes consist of 3 steps: **Step 1:** Barriers and facilitators to the implementation of OMIs (implementation constructs) and other pertinent items extracted from the eligible studies will be listed in ‘Characteristics and findings of included studies’ tables (Tables 1-4). **Step 2:** The implementation constructs will be subdivided in either barriers or facilitators and will be categorized according to the type of stakeholder. If specified, we will also subdivide these constructs according to the type of setting, interventions, the time points for recording these outcomes, and the research design of the study from which these outcomes were extracted. **Step 3:** We will list our secondary outcomes in a separate table (Table 5)

Five anticipated exemplary tables present the characteristics and findings of the included studies (Tables 1-5). Pertinent items are defined for each of these tables.

**Table 1. Research design and response rate***

| **Study and year** | **Research design** | **Selection procedures** | **Power calculation ?** | **Response rate** |
| --- | --- | --- | --- | --- |
|  |  |  |  |  |

**Study and year:** The name of the first author and the year of the publication

**Research design:** Qualitative study: interview, focus group etc. or a quantitative study: survey, questionnaire etc.

**Selection procedures?** Describe selection procedures, e.g., randomized selection of doctors from an orthodontic society, consecutively treated patients with OMIs etc.

**Power calculation?** Was a power calculation conducted? Yes/Not reported/Unclear

**Response rate:** This statistic is calculated as: The number of stakeholders that answered the survey questions/The total number of stakeholders that was contacted to answer these questions. Present the response rate for example as: 200/1000

***Score as ‘Not reported’:** When data on this entry are not reported in the research study

***Score as ‘Not applicable’**: When the entry is not applicable for the research study

**Table 2. Stakeholders (interviewed or surveyed population) and settings ***

| **Study and year** | **Type, number, and age of the stakeholders** | **Ethnicity** | **Experience of stakeholders** | **Settings/country/**  **city** |
| --- | --- | --- | --- | --- |
|  |  |  |  |  |

**Study and year:** The name of the first author and the year of the publication

**Type, number, and age of stakeholders:** Characteristics of the participants from which research data were obtained, e.g. Indian doctors, or Japanese patients, or subgroups of stakeholders etc. Include the number and age (years and months) of the stakeholders

**Ethnicity:** Present the ethnicity of the stakeholders, e.g., Indian, Japanese etc.

**Experience of stakeholders:** Stakeholders with or without experience with the interventional procedure, e.g., patients who had already undergone treatment with OMIs or clinicians who had experience with using OMIs as anchorage devices

**Setting/country/city:** Present the setting, the country, city (or outside) of the research study, e.g., private practice or university clinic in Milan, Italy etc.

***Score as ‘Not reported’:** When data on this entry are not reported in the research study

***Score as ‘Not applicable’**: When the entry is not applicable for the research study

**Table 3. Interventions***

| **Study and year** | **Definition interventions?** | **‘Specified’ or ‘Non specified’ intervention** | **Plates?** | **Type and number of OMIs** | **Location?** | **Duration (weeks)** |
| --- | --- | --- | --- | --- | --- | --- |
|  |  |  |  |  |  |  |

**Study and year:** The name of the first author and the year of the publication

**Definition interventions:** Did the author(s) present a definition of the interventional procedure? Yes/Not reported/Unclear

**‘Specified’ or ‘Non specified’ interventions:** Describe the interventional procedures. ‘***Specified’ interventions:*** these interventions refer to a specific phase or type of the interventional procedure. Phases of the intervention refer to the: anesthetics, implant insertion, orthodontic treatment with OMIs, implant removal, or the healing phase. Types of interventions refer to the: implant type and dimensions, number of implants, use of plates, the surgical procedure, implant location, timing and forces of orthodontic loading etc. [41]. ***‘Non specified’ interventions:*** these interventions refer to “any orthodontic treatment with OMIs”. Additional information on the specific phase or type of the interventional procedure is not provided

**Plates:** Were plates connected to the OMIs? Yes/Not reported/Unclear

**Type and number of OMIs:** Describe the type and number of the OMIs, e.g., 2 Quattro**implants diameter 1.5 mm and length 9 mm (**PSM Medical Solutions; Tuttlingen, Germany)

**Location:** Describe the implant insertion site, e.g., palate

**Duration:** Duration of total treatment time with OMIs in weeks

***Score as ‘Not reported’:** When data on this entry are not reported in the research study

***Score as ‘Not applicable’**: When the entry is not applicable for the research study

**Table 4. Outcomes***

| **Study and year** | **Barriers and stakeholders** | **Facilitators and stakeholders** | **Definition of the barriers and facilitators** | **Prevalence of the barrier or facilitator** | **Time point of measuring outcomes** |
| --- | --- | --- | --- | --- | --- |
|  |  |  |  |  |  |

**Study and year:** The name of the first author and the year of the publication

**Barriers and stakeholders:** List all identified barriers to the implementation of OMIs in clinical practice and list the pertinent stakeholders to which these barriers apply, e.g. patients, or clinicians etc.

**Facilitators and stakeholders:** List all identified facilitators to the implementation of OMIs in clinical practice and list the pertinent stakeholders to which these facilitators apply, e.g. patients, or clinicians etc.

**Definition of the barriers and facilitators:** Were barriers and facilitators defined by the author(s) of the eligible research study? Yes/Not reported/Unclear

**Prevalence of the barrier or facilitator:** This statistic is calculated as:

*The number of stakeholders that scored a particular construct as a barrier or facilitator to the implementation of OMIs in clinical practice/The total number of stakeholders that scored on the role of this particular construct as a barrier or facilitator to the implementation of OMIs in clinical practice*

This prevalence will be presented for example as: 30/50.

**Time point of measuring outcomes:** Outcomes are either scored prior to the intervention, immediately (within 2 weeks) or long-term (more than 2 weeks) after the interventional procedure.

***Score as ‘Not reported’:** When data on this entry are not reported in the research study

***Score as ‘Not applicable’**: When the entry is not applicable for the research study

**Table 5. Use of OMIs by clinicians***

| **Study and year** | **Research design** | **Response rate** | **Prevalence of clinicians that do not use OMIs** | **Additional information on the use by clinicians of OMIs** |
| --- | --- | --- | --- | --- |
|  |  |  |  |  |

**Study and year:** The name of the first author and the year of the publication

**Research design:** Qualitative study: interview, focus group etc. or a quantitative study: survey, questionnaire etc.

**Response rate:** This statistic is calculated as:

*The number of stakeholders that answered the survey questions/The total number of stakeholders that was contacted to answer these questions*.

Present the response rate for example as: 200/1000

**Prevalence of clinicians that do not use OMIs:** This statistic is calculated as:

*The number of clinicians that do not use OMIs/The total number of surveyed clinicians that reported on the use of OMIs in clinical practice*

**Additional information on the use of OMIs by clinicians:** Information that could give further insights in the understanding of the knowledge-to-action gap, e.g., the number of implants placed per clinician per year.

***Score as ‘Not reported’:** When data on this entry are not reported in the research study

***Score as ‘Not applicable’**: When the entry is not applicable for the research study
