# Supplementary material for: Barriers and facilitators to the implementation of orthodontic mini-implants in clinical practice: a protocol for a systematic review and meta-analysis
Source: Syst Rev. 2016 Feb 5;5:22. doi: 10.1186/s13643-016-0198-4 (PMC4743120; doi:10.1186/s13643-016-0198-4)
Supplement: Additional file 4: — Critical appraisal tools. (DOCX 17 kb) [file 13643_2016_198_MOESM4_ESM.docx]

**Additional file 4. Critical appraisal tools**

**Table 1. Joanna Briggs Qualitative Assessment and Review Instrument (JBI QARI)[30]* ****

| **Appraisal questions** | **Yes** | **No** | **Unclear** | **Not applicable** | **Comments** |
| --- | --- | --- | --- | --- | --- |
| 1)Is there congruity between the stated philosophical perspective and the research methodology ? |  |  |  |  |  |
| 2)Is there congruity between the research methodology and the research question or objectives ? |  |  |  |  |  |
| 3)Is there congruity between the research methodology and the methods used to collect data ? |  |  |  |  |  |
| 4)Is there congruity between the research methodology and the representation and analysis of data ? |  |  |  |  |  |
| 5)Is there congruity between the research methodology and the interpretation of results ? |  |  |  |  |  |
| 6)Is there a statement locating the researcher culturally or theoretically ? |  |  |  |  |  |
| 7)Is the influence of the researcher on the research, and vice-versa, addressed ? |  |  |  |  |  |
| 8)Are participants, and their voices, adequately represented ? |  |  |  |  |  |
| 9)Is the research ethical according to current criteria or, for recent studies, and is there evidence of ethical approval by an appropriate body ? |  |  |  |  |  |
| 10)Do the conclusions drawn in the research report flow from the analysis, or interpretation, of the data ? |  |  |  |  |  |

* Critical appraisal scores: ☺ Yes ☹ No ? Unclear NA Not Applicable

** Locate where information on each item can be found, e.g., Page 12 column 3.

**Table 2. Tabular presentation of the scores of the Joanna Briggs Institute Qualitative Assessment and Review Instrument (JBI QARI)[30]***

| **Study** | Q1 | Q2 | Q3 | Q4 | Q5 | Q6 | Q7 | Q8 | Q9 | Q10 |
| --- | --- | --- | --- | --- | --- | --- | --- | --- | --- | --- |
| **Author(s) ref** |  |  |  |  |  |  |  |  |  |  |

* Critical appraisal scores: ☺ Yes ☹ No ? Unclear NA Not Applicable

**Table 3. The Joanna Briggs Institute critical appraisal tool of prevalence and incidence data [31,62]* ****

| **Appraisal questions** | **Yes** | **No** | **Unclear** | **Not applicable** | **Comments** |
| --- | --- | --- | --- | --- | --- |
| 1)Was the sample representative of the target population ? |  |  |  |  |  |
| 2)Were study participants recruited in an appropriate way ? |  |  |  |  |  |
| 3)Was the sample size adequate ? |  |  |  |  |  |
| 4)Were the study subjects and the setting described in detail ? |  |  |  |  |  |
| 5)Was the data analysis conducted with sufficient coverage of the identified sample ? |  |  |  |  |  |
| 6)Were objective, standard criteria used for the measurement of the condition ? |  |  |  |  |  |
| 7)Was the condition measured reliably ? |  |  |  |  |  |
| 8)Was there appropriate statistical analysis ? |  |  |  |  |  |
| 9)Are all important confounding factors/subgroups/differences identified and accounted for ? |  |  |  |  |  |
| 10)Were subpopulations identified using objective criteria ? |  |  |  |  |  |

* Critical appraisal scores: ☺ Yes ☹ No ? Unclear NA Not Applicable

** Locate where information on each item can be found, e.g., Page 12 column 3.

**Table 4. Tabular presentation of the scores of the Joanna Briggs Institute critical appraisal tool of prevalence and incidence data [31,62]***

| **Study** | Q1 | Q2 | Q3 | Q4 | Q5 | Q6 | Q7 | Q8 | Q9 | Q10 |
| --- | --- | --- | --- | --- | --- | --- | --- | --- | --- | --- |
| **Author(s) ref** |  |  |  |  |  |  |  |  |  |  |

* Critical appraisal scores: ☺ Yes ☹ No ? Unclear NA Not Applicable
