# Supplementary material for: Barriers and facilitators to the implementation of orthodontic mini-implants in clinical practice: a protocol for a systematic review and meta-analysis
Source: Syst Rev. 2016 Feb 5;5:22. doi: 10.1186/s13643-016-0198-4 (PMC4743120; doi:10.1186/s13643-016-0198-4)
Supplement: Additional file 5: — Heterogeneity. Sources of heterogeneity and strategies for dealing with heterogeneity. (DOCX 21 kb) [file 13643_2016_198_MOESM5_ESM.docx]

**Additional file 5. Heterogeneity**

**Sources of heterogeneity**

Potential sources of heterogeneity in the eligible research studies that could influence outcomes are divided in 3 main categories: methodological, clinical, and other sources of heterogeneity [65,70]. A series of these sources are defined a priori under here. We will consult these variables to explore heterogeneity in outcomes.

**Methodological heterogeneity**

Methodological heterogeneity refers to differences in outcomes within and between studies as a result of the design, conduct and analysis, and size of the study [65,66,70]. We will consider the following potential sources of methodological heterogeneity (Table 1).

**Table 1. Potential sources of methodological heterogeneity**

| **Potential sources of methodological heterogeneity** | **Description of pertinent subgroups for each domain** |
| --- | --- |
| Research design | ***Surveys or questionnaires, interviews, and focus groups etc.*** |
| Conduct and analysis of the study | ***The risk of selection, performance, detection, attrition, and reporting bias*** [66]. |
| Study size | ***‘Study size’*** will be explored, because smaller studies often lack power and their methodological rigour tends to be suboptimal [72,73,74]. |

**Clinical heterogeneity**

Clinical heterogeneity refers to differences in outcomes within and between studies as a result of specific characteristics of stakeholders, interventions, outcomes, and setting [65,70]. We will consider the following potential sources of clinical heterogeneity (Table 2).

**Table 2. Potential sources of clinical heterogeneity**

| **Potential sources of clinical heterogeneity** | **Description of pertinent subgroups for each domain** |
| --- | --- |
| Characteristics of stakeholders | ***Ethnicity, sex, age, previous experience with OMIs etc.*** |
| Type of interventions | ***Specified interventions:*** this type of intervention refers to a specific phase or type of the interventional procedure. Phases of the intervention refer to the: anesthetics, implant insertion, orthodontic treatment with OMIs, implant removal, or the healing phase. Types of interventions refer to the: implant type and dimensions, number of implants, use of plates, the surgical procedure, implant location, timing and forces of orthodontic loading [41].  ***‘Non specified’ interventions:*** this type of intervention refers to “any orthodontic treatment with OMIs”. Additional information on the specific phase or type of the interventional procedure is not provided by the authors. |
| Outcomes | ***Pre-intervention recordings***, i.e., recordings of outcomes prior to the interventional procedure.  ***Immediate post-intervention recordings***, i.e., recordings of outcomes within 2 weeks after the completion of the interventional procedure.  ***Long-term post-intervention recordings***, i.e., recordings of outcomes after more than 2 weeks after the completion of the interventional procedure. |
| Setting | ***Private practice and university setting*** |

**Other sources of heterogeneity**

Other sources of heterogeneity refers to differences in outcomes within and between studies as a result of scientific misconduct, funding of the research study, cost of the interventional procedure, and conflicts of interest [70]. We will consider the following potential ‘other’ sources of heterogeneity (table 3).

**Table 3. Potential ‘other’ sources of heterogeneity**

| **Potential ‘other’ sources of heterogeneity** | **Description of pertinent subgroups for each domain** |
| --- | --- |
| **‘Other’ sources of heterogeneity** | ***Scientific misconduct, funding of the research study, cost of the interventional procedure, conflicts of interest etc.*** |

**Strategies for dealing with heterogeneity**

We will create forest plots to visualize statistical heterogeneity [68]. We will test the presence of statistical heterogeneity by obtaining a p-value from the comparison of Cochran’s Q with a Chi^2^ distribution with *k*−1 degrees of freedom (*k* = the number of eligible studies) [75-77]. We will take in consideration that the power of this test is low when the number of eligible studies is low and is excessive when the numbers of studies is high and particularly when study sizes are large [75,77].

We will calculate Kendall’s Tau^2^ for estimating the variance of true effect sizes between studies and Tau as the standard deviation of this estimate [68,75,77-80]. These estimates will be used to represent the magnitude of the heterogeneity [75,79]. I^2^ will also be calculated and reflects the proportion of the between study variance to the total variance across the observed effect estimates [75,77,79]. The interpretation of this measure of inconsistency will be conducted according to the guidelines described in the Cochrane Handbook for Systematic Reviews of Interventions, i.e., I^2^: 0% to 40%: might not be important; I^2^: 30% to 60%: may represent moderate heterogeneity; I^2^: 50% to 90%: may represent substantial heterogeneity; and I^2^: 75% to 100%: considerable heterogeneity [65].

If clarification on issues of heterogeneity is indicated, we will contact authors according to our protocol (Additional file 1). Besides assessing the role of ‘a priori’ defined sources of heterogeneity we will also explore whether additional (‘post hoc’) sources of heterogeneity could be identified in the eligible studies. We will weigh the various strategies for dealing with statistical heterogeneity according to the Cochrane Handbook for Systematic Reviews of Interventions [65].

1. Double-check whether the data extraction from the eligible studies and the data entry into the Comprehensive Meta-Analysis (CMA) program were done correctly [68].
2. Assess whether pooling of outcomes is indicated based on our criteria for conducting a quantitative synthesis.
3. Investigate potential causes of heterogeneity through subgroup analyses and meta-regression.
4. Ignore heterogeneity by applying a fixed-effect meta-analysis.
5. Investigate heterogeneity that cannot be explained through a random-effects meta-analysis.
6. Change the measure of effect size.
7. Exclude studies, but consider the consequences of introducing bias.
